# Correlation Analysis of *BLTP1* (*KIAA1109*) and *KIF27* Gene Polymorphisms with Wool Traits in Subo Merino Sheep

**DOI:** 10.3390/genes17030295

**Published:** 2026-02-28

**Authors:** Qingfa Yan, Sen Tang, Asma Anwar, Gvlnigar Amar, Yaqian Wang, Wenna Liu, Cuiling Wu, Xuefeng Fu

**Affiliations:** 1 Laboratory for the Conservation and Regulatory Biology of Special Environmental Species in Xinjiang, Key Laboratory of Special Environment Biodiversity Application and Regulation in Xinjiang, College of Life Science, Xinjiang Normal University, Urumqi 830017, China; v2762636615@163.com (Q.Y.); 13579812147@163.com (G.A.); 2 Xinjiang Key Laboratory of Animal Biotechnology, Xinjiang Uygur Autonomous Region Academy of Animal Science, Urumqi 830011, China; tangsensen610@163.com (S.T.); asma247462@163.com (A.A.); wangyaqlan@163.com (Y.W.); lwn2362@163.com (W.L.); 3 Xinjiang Key Laboratory of Reproductive Regulation and Breeding for Ruminant Livestock, Xinjiang Uygur Autonomous Region Academy of Animal Science, Urumqi 830011, China; 4 Key Laboratory of Genetic Breeding and Reproduction of Her-Bivorous Livestock of Ministry of Agriculture and Rural Affairs, Xinjiang Uygur Autonomous Region Academy of Animal Science, Urumqi 830011, China; 5 Key Laboratory of Genetics Breeding and Reproduction of Xinjiang Wool-Sheep & Cashmere-Goat (XJYS1105), Xinjiang Uygur Autonomous Region Academy of Animal Science, Urumqi 830011, China

**Keywords:** Subo Merino sheep, wool traits, *BLTP1*, *KIF27*, genetic polymorphism

## Abstract

**Background/Objectives**: The Subo Merino sheep is a high-quality fine-wool breed developed through progressive hybridization, characterized by high wool yield and excellent wool quality. This study is designed to investigate the effects of two gene polymorphisms in Subo Merino sheep on wool traits, thereby providing critical theoretical and technical support for the breeding of high-quality fine-wool sheep. **Methods**: In this study, 944 one-year-old Subo Merino sheep were genotyped for coding regions of the *BLTP1* and *KIF27* genes using the Fluidigm BioMark™ HD system. Association between SNP loci and wool traits was analyzed via the least squares means method in SAS 9.4. Protein–protein interaction networks were constructed using the STRING database, and protein structures before and after mutation were predicted with SOPMA and SWISS-MODEL. **Results**: The results revealed that *BLTP1* gene identified a missense mutation site SNP1, which resulted in a nucleotide change c.812 (C > T) and an amino acid change p.Pro271Leu. *KIF27* gene identified a missense mutation site SNP2, which resulted in a nucleotide change c.3896 (T > C) and an amino acid change p.Met1299Thr. Association analysis showed that SNP1 had a significant effect on wool crimp number (CN) and staple length (SL) (*p* < 0.05), while SNP2 significantly affected live weight after shearing (LWAS) (*p* < 0.05). Protein structure prediction showed that mutations at SNP1 and SNP2 primarily led to changes in α-helix, extended chain, and random coil structures. **Conclusions**: These results suggest that SNP1 in *BLTP1* and SNP2 in *KIF27* could serve as potential molecular markers for wool traits in Subo Merino sheep. This study provides theoretical support and candidate gene targets for molecular marker-assisted breeding, contributing to genetic improvement and efficient breeding of this fine-wool breed.

## 1. Introduction

China is one of the largest countries in sheep farming, but there remains a significant gap in the domestic processing demand for fine-wool [1]. The Chinese wool textile industry relies on imports for nearly half of its wool supply, with a particularly heavy dependence on imports for ultra-fine wool fibers measuring less than 19.5 microns in diameter. This underscores Chinese substantial market demand for high-quality wool, especially ultra-fine varieties [2].

Subo Merino Sheep are an ultra-fine-wool sheep breed developed in China in 2014 through strict phenotypic selection and genetic evaluation, after three breeding stages [3,4]. The wool diameter of Subo Merino Sheep is precisely controlled between 17.0~19.0 μm, with stable genetic performance, ensuring the continued transmission of excellent traits and reducing the gap between domestic and imported wool [5]. Exploring molecular markers related to wool traits in Subo Merino Sheep provides theoretical support for the utilization of this breed’s excellent genetic resources [6]. Wool traits are quantitative traits controlled by multiple minor-effect genes. Identifying genetic markers for these traits provides theoretical and technical support for molecular breeding in fine-wool sheep, ultimately enhancing wool production efficiency and quality—thereby boosting the self-sufficiency and market competitiveness of Chinese wool industry and delivering significant economic value.

Based on the results of the previous genome-wide association study conducted by the research team, it was suggested that the *BLTP1* and *KIF27* genes are associated with wool traits [7]. Related studies have shown that *BLTP1* is an important non-vesicular lipid transport protein in multiple species [8]. In eukaryotic cells, the bridge lipid transport protein *BLTP1* can promote the exchange of various lipids between the membranes of adjacent organelles through membrane contact sites, playing a crucial role in lipid metabolism, membrane transport, and cell signal transduction processes [9,10]. This mechanism is essential for the effective turnover and distribution of various intracellular lipids, such as phospholipids [8,9,10]. Studies on transmembrane proteins reveal that proteins in the TMEM170 family, acting as endoplasmic reticulum lipid flippases, can work synergistically with *BLTP1* to form molecular bridges that mediate bulk lipid transport, ensuring the efficient flow of lipids between organelles [11]. Membrane contact sites are important locations for intracellular lipid metabolism and signal transduction, and *BLTP1* regulates the function of these sites, making it a key component in maintaining material exchange and signal integration between organelles [9]. In *Caenorhabditis elegans*, the protein *LPD-3* (the human homolog of *BLTP1*) constructs a molecular bridge between the endoplasmic reticulum and the plasma membrane, mediating lipid transport, and is crucial for the organism’s cold resistance [12]. In summary, *BLTP1* has been confirmed in multiple species to be involved in lipid transport and metabolism processes, and its function is highly conserved in evolution. This key characteristic supports the hypothesis that *BLTP1* may be an important candidate gene affecting wool traits by regulating lipid metabolism pathways.

*KIF27* belongs to the kinesin-4 subfamily of motor proteins and is a key molecule in regulating cell mitosis and ciliogenesis. Recent studies have shown that *KIF27* plays a central role in the formation and maintenance of cilia. Cilia play important roles in the physiological processes of mammalian nervous, respiratory, and reproductive systems [13,14]. Furthermore, *KIF27* affects microtubule dynamics, suggesting its conserved role in microtubule organization within the kinesin-4 family [15]. Members of the kinesin protein family are generally involved in the intracellular transport of various “cargoes” along microtubules, including vesicles, protein complexes, and organelles [16]. In *KIF27* and *KIF7*, changes in the chemical–mechanical coupling lead to impaired motility, and this unique motility property may correspond to their specific regulatory roles in the Hedgehog signaling pathway and ciliogenesis [15,17]. Moreover, abnormal expression and dysfunction of kinesin family genes are associated with various diseases [16]. In a study on the polymorphism of the *KIF-I* gene in cashmere goats, an SNP in exon 1 was found to be significantly related to fleece density [18]. In summary, functional variations in the kinesin family member *KIF27* may influence the growth and development of wool traits from multiple dimensions.

Early studies have shown a significant genetic correlation between wool length and fineness in Subo Merino Sheep (r_A_ = 0.388), meaning that selecting for finer wool often leads to a reduction in wool length and clean fleece weight. This phenomenon has a stronger effect on live weight and clean fleece weight after shearing (r_A_ = 0.616), where selecting for finer wool leads to a decrease in clean fleece weight, indirectly resulting in a reduction in live weight after shearing [4]. This antagonistic relationship between traits restricts further improvement of the breed. Therefore, analyzing the polymorphism distribution of the *BLTP1* and *KIF27* genes in the Subo Merino Sheep population and evaluating their association with wool fineness, length, crimp, and other phenotypes will not only help elucidate the molecular mechanisms of ultra-fine-wool formation but also provide a theoretical basis for designing high-precision molecular markers. This will be of significant importance for early selection and accelerating genetic progress in fine-wool sheep breeding.

## 2. Materials and Methods

### 2.1. Sample Collection

This study used 944 one-year-old female Subo Merino sheep, this variety is seasonally propagated. The animals were sourced from two locations: 473 from the Gongnaisi Breeding Farm in Xinjiang Yili Prefecture (*n* = 473) and 471 from the Baicheng Breeding Farm in Aksu Prefecture, Xinjiang (*n* = 471). At both farms, the sheep were managed under a similar dietary regimen involving a combination of grazing and supplementary feeding. In May, blood samples (5 mL) were collected from the jugular vein of each sheep into anticoagulant tubes. The collected blood was mixed and stored at −20 °C for subsequent genomic DNA extraction. Additionally, wool samples were collected from each sheep at a point 10 cm above the posterior edge of the left scapula along the midline. The following wool traits were directly measured: greasy fleece weight (GFW), live weight before shearing (LWBS), and live weight after shearing (LWAS).

The environmental conditions during the measurement were: temperature (20 ± 2 °C) and humidity (65% ± 4%). The following wool traits were measured using the Optical Fiber Diameter Analyzer OFDA2000 (OFDA-2000BT, BSC Electronics, Perth, Australia): mean fiber diameter (MFD), staple length (SL), hair length (HL), Fineness Count (FC), coefficient of variation in fiber diameter (CVFD), fiber diameter standard deviation (FDSD), and crimp and crimp number (CN).

### 2.2. SNP Genotyping and Statistical Analysis

This study utilized the Fluidigm BioMark™ HD system (BioMark™ HD, San Francisco, CA, USA) to perform SNP genotyping for the *BLTP1* and *KIF27* genes in 944 individual sheep. The SNP genotype frequencies, allele frequencies, observed heterozygosity (Ho), effective allele number (Ne), expected heterozygosity (He), polymorphism information content (PIC), and Hardy–Weinberg equilibrium (HWpval) were calculated using Popgene 1.32 software.

The correlation between different SNP genotypes and wool traits was analyzed using SAS 9.4 software. The results are presented as least squares means ± standard errors. The linear model used was:

Yick = μ + Gi + Fc + eick

In the formula, Yick denotes the phenotypic value of an individual fine-wool sheep; μ denotes the population mean; Gi denotes the genotype SNP effect; Fc denotes the farm effect; and eick denotes random error.

### 2.3. Protein Structure Prediction and Network Interaction Analysis

Based on the sequence information of the target genes *BLTP1* (ENSOART00000000336.1) and *KIF27* (ENSOART00000009775.1) from the Ensembl database, the gene sequences were translated into amino acid sequences using the NOVOPRO website. The secondary protein structure was predicted using SOPMA “https://npsa.lyon.inserm.fr/cgi-bin/npsa_automat.pl?page=/NPSA/npsa_sopma.html” (accessed on 12 May 2025) and the tertiary protein structure was predicted using SWISS-MODEL “https://swissmodel.expasy.org/” (accessed on 14 May 2025). Based on protein interaction data from the STRING database, interaction networks were constructed with *BLTP1* and *KIF27* as the core nodes.

## 3. Results

### 3.1. Descriptive Statistics of Wool Traits

Descriptive statistical analysis of 11 wool traits (MFD, FDSD, CVFD, SL, FC, Crimp, SL, CN, GFW, LWBS, and LWAS) in Subo Merino Sheep was performed using SAS 9.4 software, and outliers were removed (Table 1). The data are presented as mean ± standard deviation. The analysis results show that the trait data align with objective facts, with the small standard deviation indicating that the variable distribution is relatively concentrated around the mean.

### 3.2. SNP Mutation Information Statistics

SNP genotyping of the two mutation sites, SNP1 in the *BLTP1* gene and SNP2 in the *KIF27* gene, was successfully performed on 944 Subo Merino Sheep using Fluidigm technology. After aligning with the reference gene sequence, SNP1 was identified as a missense mutation in exon 7 of the *BLTP1* gene, resulting in the nucleotide change c.812 (C > T) and causing the amino acid change p.Pro271Leu. SNP2 was identified as a missense mutation in exon 18 of the *KIF27* gene, resulting in the nucleotide change c.3896 (T > C) and causing the amino acid change p.Met1299Thr (Table 2).

### 3.3. SNP Genetic Polymorphism Analysis

This genotype frequencies, allele frequencies, observed heterozygosity (Ho), expected heterozygosity (He), and polymorphism information content (PIC) of the *BLTP1* SNP1 and *KIF27* SNP2 loci in Subo Merino Sheep are shown in Table 3. For *BLTP1* SNP1, the genotypes AA, GA, and GG were identified, with GA (0.471) being the dominant genotype. For *KIF27* SNP2, the genotypes TT, TC, and CC were identified, with TC (0.469) being the dominant genotype. The PIC for SNP1 was 0.354 and for SNP2 was 0.374, both of which are considered moderate polymorphism. The He for both SNP1 and SNP2 was close to 0.5, indicating that the genetic diversity at these two loci is at a moderately high level, with SNP2 showing slightly higher diversity. Both SNP loci also conform to Hardy–Weinberg equilibrium (*p* > 0.05), indicating that the studied population is in random mating at these loci.

### 3.4. Correlation Analysis Between SNPs and Wool Traits

The correlation between the SNP1 locus in the *BLTP1* gene and the SNP2 locus in the *KIF27* gene with wool traits in Subo Merino Sheep was analyzed using SAS 9.4 software. The results showed that the *BLTP1* gene SNP1 locus was significantly associated with CN (*p* < 0.05) and SL (*p* < 0.05), but not with other phenotypes such as FC, MFD, FDSD, and MSL (*p* > 0.05). The *KIF27* gene SNP2 locus showed a significant association with LWAS (*p* < 0.05), but no significant association with other traits (*p* > 0.05) (Table 4).

### 3.5. Protein Structure Prediction Analysis

The secondary protein structure before and after mutation was predicted using the SOPMA website, while the tertiary structure was predicted using the SWISS-MODEL website. A comparison of the protein secondary structure of the C and T base sequences for *BLTP1* gene SNP1 is shown in Table 5. The change in the base at the SNP1 locus led to an increase in the α-helix content from 23.13% before mutation to 23.73% after mutation, an increase in the extended chain from 10.91% to 11.38%, while the random coil decreased from 65.96% to 64.89%. For *KIF27* gene SNP2, a comparison of the T and C base sequences of the protein secondary structure is shown in Table 5. After the mutation at SNP2, the α-helix content increased from 75.63% to 75.84%, while the extended chain decreased from 4.95% to 4.87%, and the random coil decreased from 19.43% to 19.28% after the mutation.

**Figure 1:**
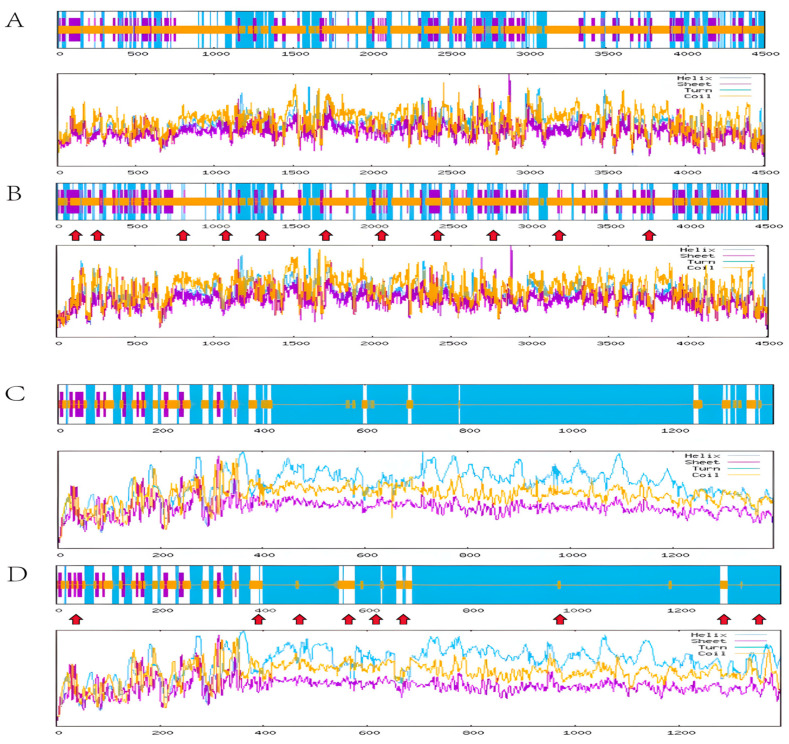
Comparison of predicted protein secondary structures before and after SNP mutations. (**A**) Secondary Structure Prediction of Before the SNP1 Mutation, (**B**) Secondary structure prediction of after the SNP1 mutation, (**C**) Secondary Structure Prediction of Before the SNP2 Mutation, (**D**) Secondary structure prediction of after the SNP2 mutation. The red arrow indicates the change after the mutation.

The missense mutations in genes cause changes in amino acid residues, so the protein secondary structure before and after mutation was predicted using the SOPMA v.2 website. A comparison of the protein secondary structure of the C and T base sequences at the *BLTP1* gene SNP1 locus is shown in Figure 1. The SNP1 locus causes the amino acid change p.Pro271Leu, and the secondary structure changes in 11 main regions, with overall significant changes that are not concentrated in one area. The changes occur between amino acids 0–4000 (Figure 1B). For *KIF27* gene SNP2, a comparison of the T and C base sequences of the protein secondary structure is shown in Figure 1. The SNP2 locus causes the amino acid change p.Met1299Thr, with changes occurring in 9 main regions. These changes are concentrated in the 0–1400 amino acid range (Figure 1D).

**Figure 2:**
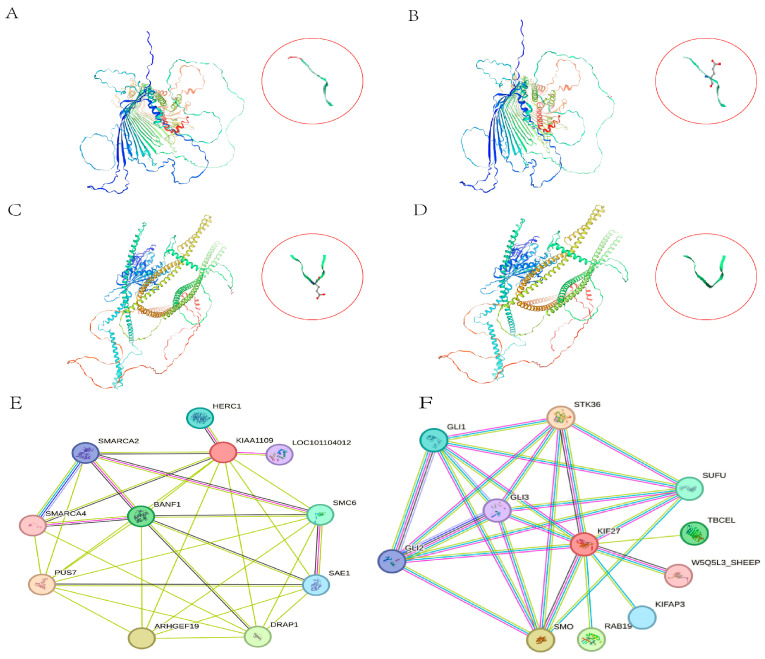
Protein–Protein Interaction Network of BLTP1 and KIF27 and Tertiary Structure Prediction Before and After SNP1 and SNP2 Mutations. (**A**) The predicted tertiary structure of BLTP1 prior to the SNP1 mutation. (**B**) The predicted tertiary structure of the BLTP1 protein following the SNP1 mutation. (**C**) The predicted tertiary structure of KIF27 prior to the SNP2 mutation. (**D**) The predicted tertiary structure of the KIF27 protein following the SNP2 mutation. (**E**) The protein interaction networks for BLTP1. (**F**) The protein interaction networks for KIF27.

Through observation of the tertiary structures of *BLTP1* (Figure 2A,B) and *KIF27* (Figure 2C,D), it can be seen that the mutations at SNP1 and SNP2 have a minimal impact on the overall shape of the protein’s tertiary structure, with no significant structural changes. However, localized changes were observed at specific nodes. Protein interaction network analysis revealed that several key potential genes, such as *SMARCA2*, *SMARCA4*, and *BANF1*, play crucial roles in the *BLTP1* network topology (Figure 2E). Additionally, *KIF27* interacts with potential genes including *STK36*, *GLI1*, *GLI2*, *GLI3*, *SUFU*, and *SMO* (Figure 2F).

## 4. Discussion

Currently, numerous scholars are conducting extensive research on molecular markers for wool traits using methods such as candidate gene approaches, QTL mapping, and genome-wide association studies. These studies have accumulated valuable experimental data and theoretical insights for the molecular breeding of fine-wool sheep [19,20,21]. Wool traits (such as wool diameter, wool yield, and wool length) are quantitative traits, controlled by multiple genes with small effects. Therefore, the discovery of genetic markers for these traits requires further in-depth investigation. In this study, based on our previous research, we selected the *BLTP1* and *KIF27* genes and performed polymorphism identification in the Subo Merino Sheep population. The results successfully genotyped SNP1 (c.812C > T, p.Pro271Leu) in *BLTP1* and SNP2 (c.3896T > C, p.Met1299Thr) in *KIF27*, and both SNP loci were in Hardy–Weinberg equilibrium (*p* > 0.05). Phenotypic association analysis indicated that SNP1 and SNP2 were significantly associated with key wool traits. Although this study did not systematically explore the entire genome, our findings still provide valuable clues for the functional study of these genes. More importantly, the results fill the research gap of *BLTP1* and *KIF27* genes in Subo Merino sheep, a unique, high-quality fine-wool breed, and provide a reference for the molecular marker-assisted breeding technology of fine-wool sheep.

This study found a significant association between the SNP1 mutation in the *BLTP1* gene and SL as well as CN in Subo Merino Sheep (*p* < 0.05). Previous studies have shown that in Chinese Merino sheep, live weight after shearing is moderately positively genetically correlated with staple length, and CN is negatively genetically correlated with MFD [22,23,24]. However, in our study, no similar trends were observed between the genotypes of SNP1. Specifically, the GA genotype showed higher live weight before shearing compared to the GG and AA genotypes. Although this difference did not reach statistical significance, it provides a reference for multi-trait synergistic improvement. We are also focused on identifying molecular genetic markers that can simultaneously improve both wool traits and growth performance in sheep, to provide theoretical support for the breeding of dual-purpose sheep (both wool and meat).

*BLTP1*, a member of the bridge lipid transfer protein family, primarily functions in lipid transport, maintaining the dynamic balance of organelle membranes, and is implicated in the pathogenesis of certain diseases [8]. In mouse models, the deletion of *BLTP1* affects the survival and normal development of the neuromuscular junction, indicating the gene’s importance in the development and functional maintenance of the nervous system [10]. Case studies have shown that variations in the splicing sites of the *BLTP1* gene can lead to multiple congenital malformations in fetuses, highlighting *BLTP1*’s indispensable role in mammalian development. Dysfunction in *BLTP1* could result in severe congenital defects [25].

Currently, there is limited research on the role of the *BLTP1* gene in hair follicle growth and development, but lipid transport plays a crucial role in maintaining the homeostasis of hair follicle cells [26,27]. Studies in mice have shown that immature dermal adipocytes and mature adipocytes play different roles in hair follicle development and hair growth [28,29]. In our previous research using transcriptomics and proteomics, we found that lipid-related genes are associated with the cyclical nature of secondary hair follicles in the Jiangnan cashmere goat. Cell experiments revealed that *FA2H* promotes the proliferation of secondary hair follicle dermal papilla cells, with *FA2H* encoding a fatty acid 2-hydroxylase [30]. *PLIN2*, a gene that regulates lipid metabolism, when overexpressed, can modulate the proliferation and apoptosis of DPCs, affecting the hair follicle growth cycle and cashmere fiber production [31]. Moreover, protein interaction network analysis showed that *BLTP1* directly interacts with *SMARCA2*, *SMARCA4*, and *BANF1*. Notably, *SMARCA4* and *SMARCA2* are key pathogenic genes in Coffin–Siris syndrome, while pathogenic variants in both alleles of *BANF1* cause autosomal recessive Néstor–Guillermo progeroid syndrome, both of which lead to sparse hair growth [32,33]. Combining these findings with our results, a novel insight is proposed: *BLTP1* may regulate the proliferation and apoptosis of hair follicle dermal papilla cells by participating in lipid transport, and further interact with *SMARCA2*, *SMARCA4*, and *BANF1* to affect hair follicle development, thereby regulating wool staple length and crimp number. Although the mechanism by which the SNP1 mutation in *BLTP1* affects staple length and crimp number remains unclear, the above results suggest that *BLTP1* may be involved in the regulation of hair follicle development and hair growth. The specific regulatory mechanisms will be further explored in our future research.

In this study, we found that the SNP2 mutation in the *KIF27* gene was significantly associated with LWAS in Subo Merino Sheep (*p* < 0.05). *KIF27* belongs to the kinesin-4 superfamily, and its members play key roles in intracellular material transport, microtubule dynamics, and the maintenance of ciliogenesis functions [15,16,17]. Notably, in the protein interaction network, *KIF27* interacts with genes annotated in the Sonic Hedgehog (Shh) signaling pathway, including *STK36*, *GLI1*, *GLI2*, *GLI3*, *SUFU*, and *SMO*. This suggests that *KIF27* may play an important role in the Hedgehog (Hh) signaling pathway in mammals. The Hh pathway, particularly Sonic Hedgehog is a core signaling pathway that regulates hair follicle development. Research on Shh has shown that when the *SMO* gene is specifically deleted in cells, Shh no longer affects hair follicles, adipocytes, and endothelial cells. Instead, Shh directly acts on adipocyte precursors to induce dermal fat generation [34]. Early studies have also confirmed that Shh is essential for the morphogenesis of hair follicles in mouse embryos [35], and continues to play a role in the cyclical regeneration of hair follicles [36]. The regulation of *Gli2* by *SUFU* and *SPOP* is crucial for the differentiation of cochlear hair cells in mammals, and SUFU is a negative regulator of Shh signaling, controlling the timing and progression of hair cell differentiation [37]. In hair follicles, the Hh signaling pathway is transmitted through primary cilia and primarily activates downstream *Gli2* transcription factors to perform its functions; however, *Gli3* also has a compensatory activation pathway that does not depend on cilia [38]. Additionally, hair follicle development requires the coordinated regulation of several signaling pathways, including Wnt signaling pathways (necessary for structural formation), Notch signaling pathways (regulating cell differentiation), and Bmp signaling pathways (a negative regulator) [39]. Therefore, as a potential component of the Hh pathway, *KIF27* may influence this pathway and contribute to the fine-tuned regulation of hair follicle development. In studies on goats, circKIF27 was found to be widely expressed in the basal layer melanocytes of the skin, with differential expression in melanocytes of white and brown skin. circKIF27 can inhibit cell proliferation, reduce tyrosinase activity, and decrease melanogenesis by targeting the miR-129-5p/TGIF-2 pathway, thus participating in goat skin color formation [40]. In our study, the SNP2 mutation in *KIF27* was significantly associated with live weight after shearing (LWAS) in Subo Merino Sheep (*p* < 0.05), but there was no significant effect on wool traits (*p* > 0.05). Although the specific mechanism by which the SNP2 mutation in *KIF27* affects live weight after shearing remains unclear, these results suggest that *KIF27* may participate in the regulation of hair follicle development and hair growth through the Shh signaling pathway. Further research is needed to better understand the precise regulatory role of *KIF27* in hair follicle development and wool growth.

From a practical perspective, the results of this study enrich the genetic marker resources of Subo Merino sheep, help protect and utilize the excellent genetic resources of local fine-wool sheep, and promote the sustainable development of the fine-wool sheep industry. It should be noted that this study still has certain limitations. Only one SNP locus was identified in each of the *BLTP1* and *KIF27* genes, and the regulatory mechanisms of these SNPs on wool traits and growth performance have not been verified by cell and animal experiments. In future research, we will further expand the sample size, screen more polymorphic loci of the *BLTP1* and *KIF27* genes, and verify the functional roles of target SNPs through in vitro cell experiments and in vivo animal experiments to clarify their specific regulatory mechanisms.

## 5. Conclusions

In this study, we identified two missense mutations: SNP1 (c.812C > T, p.Pro271Leu) in the *BLTP1* gene and SNP2 (c.3896T > C, p.Met1299Thr) in the *KIF27* gene. Both mutation loci were consistent with the Hardy–Weinberg equilibrium law in the Subo Merino sheep population. Association analysis confirmed that SNP1 significantly affected SL and CN (*p* < 0.05), while SNP2 was significantly correlated with LWAS (*p* < 0.05). Combined with protein structure prediction and protein–protein interaction network analysis, it is speculated that *BLTP1* may regulate hair follicle development through lipid transport and its interaction with *SMARCA2*, *SMARCA4*, and *BANF1*, while *KIF27* may participate in the hair follicle regulation process through the Sonic Hedgehog signaling pathway. In summary, SNP1 and SNP2 can be used as potential molecular markers for wool traits in Subo Merino sheep, providing important references for fine-wool sheep breeding and helping to shorten the breeding cycle, reduce the cost of phenotypic selection, and further improve the efficiency and accuracy of breeding.

## Figures and Tables

**Table 1:** Descriptive Statistics of Wool Properties.

Traits	N	Mean	SD	CV (%)	Range
MFD/µm	940	17.71	1.84	10.39	13.3~24.0
FDSD/µm	933	4.14	0.57	13.77	3.0~6.4
CVFD/%	941	23.38	2.24	9.58	17~30.8
SL/mm	929	88.26	11.77	13.34	65~130
FC/(count)	934	66.82	2.52	3.77	60~80
Crimp/(count)	934	1.84	0.62	33.70	0~3.0
HL/cm	933	9.89	0.95	9.61	7~14
CN/(count)	933	13.92	3.04	21.60	7~25
LWBS/kg	699	34.58	0.18	13.58	23~50
GFW/kg	699	3.39	0.02	15.76	2.0~5.6
LWAS/kg	669	34.10	0.19	14.59	20~50

**Table 2:** *BLTP1* and *KIF27* Mutation Site Information Table.

Genes	SNPs	Chromosomal Location	Mutation Site	Amino Acid Change
*BLTP1*	SNP1	17:35163382	c.812 C > T	p.Pro271Leu
*KIF27*	SNP2	2:35685765	c.3896 T > C	p.Met1299Thr

**Table 3:** The basic characteristics of 2 single nucleotide polymorphisms in 2 genes.

Genes	SNPs	Genotype	Number	Genotype Frequency	Allele Frequency	H_O_	H_E_	Ne	PIC	HWpval	X^2^
*BLTP1*	SNP1	GG	383	0.407	A (0.358) G (0.642)	0.471	0.459	1.850	0.354	0.456	0.557
		GA	443	0.471							
		AA	115	0.122							
*KIF27*	SNP2	TT	213	0.226	C (0.540) T (0.460)	0.470	0.497	1.988	0.374	0.081	3.052
		TC	442	0.469							
		CC	288	0.305							

**Table 4:** Association Analysis of SNP1 and SNP2 Mutation Sites with Wool Traits.

Traits	*BLTP1* (SNP1)			*KIF27* (SNP2)		
	GG (*n* = 383)	GA (*n* = 443)	AA (*n* = 115)	TT (*n* = 213)	TC (*n* = 442)	CC (*n* = 288)
MFD, µm	17.64 ± 0.07	17.74 ± 0.07	17.82 ± 0.13	17.68 ± 0.10	17.80 ± 0.07	17.60 ± 0.09
FDSD, µm	4.12 ± 0.03	4.17 ± 0.02	4.15 ± 0.05	4.12 ± 0.04	4.16 ± 0.02	4.15 ± 0.03
CVFD, %	23.32 ± 0.11	23.49 ± 0.10	23.20 ± 0.21	23.27 ± 0.16	23.32 ± 0.11	23.55 ± 0.13
SL, mm	**88.13 ± 0.58 ^a^**	**89.56 ± 0.54 ^b^**	**86.74 ± 1.05 ^a^**	88.23 ± 0.81	88.62 ± 0.54	89.06 ± 0.69
Crimp, count	1.87 ± 0.03	0.81 ± 0.03	1.85 ± 0.06	1.84 ± 0.04	1.83 ± 0.03	1.84 ± 0.04
FC, count	66.86 ± 0.13	66.79 ± 0.12	66.72 ± 0.23	66.82 ± 0.18	66.71 ± 0.12	66.99 ± 0.15
HL, cm	9.88 ± 0.04	9.93 ± 0.04	9.80 ± 0.08	9.95 ± 0.06	9.87 ± 0.04	9.88 ± 0.05
CN, count	**13.91 ± 0.12 ^a^**	**13.78 ± 0.11 ^b^**	**14.34 ± 0.21 ^a^**	13.84 ± 0.16	13.92 ± 0.11	13.93 ± 0.14
LWBS, kg	33.10 ± 0.21	33.27 ± 0.20	33.04 ± 0.38	32.98 ± 0.29	33.47 ± 0.19	32.85 ± 0.25
LWAS, kg	32.94 ± 0.27	32.86 ± 0.24	33.27 ± 0.47	**32.62 ± 0.33 ^a^**	**33.48 ± 0.25 ^b^**	**32.33 ± 0.32 ^a^**
GFW, kg	3.26 ± 0.03	3.29 ± 0.03	3.24 ± 0.05	3.31 ± 0.04	3.28 ± 0.03	3.23 ± 0.04

Note: Different lowercase letters in the same column indicate significant differences (*p* < 0.05); the same letters or no letters indicate no significant difference (*p* > 0.05). Significance is indicated by bold font.

**Table 5:** Effect of the SNP Mutation Site on Protein Secondary Structure.

SNPs	Base Change	α-Helix	β-Strand	Random Loop
SNP1	C	1043 (23.13%)	492 (10.91%)	2974 (65.96%)
	T	1070 (23.73%)	513 (11.38%)	2926 (64.89%)
SNP2	T	1055 (75.63%)	69 (4.95%)	271 (19.43%)
	C	1058 (75.84%)	68 (4.87%)	269 (19.28%)

## Data Availability

The original contributions presented in this study are included in the article. Further inquiries can be directed to the corresponding authors.

## Abbreviations

The following abbreviations are used in this manuscript:

MFD	Mean Fiber Diameter
FDSD	Fiber Diameter Standard Deviation
CVFD	Fiber Diameter Coefficient of Variation
SL	Staple Length
FC	Fineness Count
HL	Hair Length
Crimp	Crimp
CN	Crimp Number
GFW	Greasy Fleece Weight
LWBS	Live Weight Before Shearing
LWAS	Live Weight After Shearing
